# PPAR-γ agonist rosiglitazone ameliorates peritoneal deterioration in peritoneal dialysis rats with LPS-induced peritonitis through up-regulation of AQP-1 and ZO-1

**DOI:** 10.1042/BSR20180009

**Published:** 2018-06-21

**Authors:** Yunfang Zhang, Junxia Feng, Qi Wang, Shili Zhao, Jiaqi Xu, Hongyan Li

**Affiliations:** Department of Nephrology, Huadu District People’s Hospital, Southern Medical University, 22# Baohua Road, Huadu District, Guangzhou 510800, China

**Keywords:** AQP-1, peritonitis, peritoneal dialysis, rosiglitazone, ZO-1

## Abstract

Peritonitis is still a major cause of the death in peritoneal dialysis (PD) patients despite the significant decline of the peritonitis rates in recent years. The present study is designed to evaluate the therapeutic potential of peroxisome proliferator-activated receptor-γ agonist, rosiglitazone, on the structure and function of the peritoneum in a PD rat accompanied with peritonitis induced by lipopolysaccharide (LPS). Our data showed that the peritoneal membrane in the LPS-only group showed increased peritoneal thickness, vessel density, and hypercellularity compared with the PD-only group. Rosiglitazone administration significantly inhibited increase of the three indicators in PD rats with LPS treatment. In line with this, rosiglitazone improved function of the peritoneum in LPS-induced PD rats receiving rosiglitazone, which was reflected by decreased D/P urea and D/P albumin. Mechanistically, rosiglitazone-mediated improvements in the damaged structure and function of the peritoneum in PD rats with LPS treatment were associated with reduced inflammation and preserving mesothelial cell monolayer resulted from up-regulation of AQP-1 and ZO-1. Our findings thus suggest that peroxisome proliferator-activated receptor γ (PPAR-γ) activation might be a reasonable strategy to prevent and ameliorate peritoneal deterioration in PD patients, especially with peritonitis.

## Introduction

Peritoneal dialysis (PD) is a renal replacement strategy in patients with end-stage renal failure by using the peritoneum as a semipermeable membrane for ultrafiltration (UF) and diffusion. PD significantly lowers infection rate, improves quality of life, and increases the survival rate of patients [1]. However, the peritoneal membrane with long-term PD shows a decline in peritoneal ultrafiltration capacity or ultrafiltration failure (UFF) [2], which is often caused by the severely damaged architecture of the peritoneum during peritonitis [3].

The peritoneum is mainly composed of a single layer of mesothelial cells, which are covered by connective tissue containing a few fibroblasts, mast cells, macrophages, and vessels [4]. Accumulated evidence shows that peritoneal mesothelial cells (PMCs) play an important role in the maintenance of peritoneal homeostasis, immune surveillance, antigen presentation, inflammation, and wound healing, regulating the structure and function of the peritoneum [5,6]. However, chronic exposure to nonphysiological PD solutions and peritonitis caused by repeated microbial infection results in a loss of the mesothelial cells monolayer, submesothelial fibrosis, angiogenesis, and hyalinizing vasculopathy, which subsequently causes increased rates of small-solute transport and ultrafiltration dysfunction of the peritoneal membrane [7,8]. Inflammatory cells and myofibroblasts are considered to be the main contributor to structural and functional impairment of the peritoneum during long-term PD [9,10].

PPAR-γ or PPARG, also known as the glitazone receptor, is a ligand-activated transcription factor that plays an essential role in the regulation of fatty acid storage and glucose metabolism [11]. In addition, PPAR-γ activation has been shown anti-inflammatory effects by regulating both innate and adaptive immune responses [12]. In line with this, several studies show that PPAR-γ has antifibrotic and anti-inflammation effects in the peritoneum. For example, PPAR-γ agonist troglitazone reduced the expression of TGF-β1 and extracellular matrix accumulation in the peritoneal mesothelial cells stimulated by glucose [13]. Similarly, in our previous studies we demonstrated that the PPAR-γ activator, rosiglitazone, attenuated inflammation via NF-κB inhibition in LPS-induced peritonitis [14]. Recently, we also showed that rosiglitazone relieved the injury by inhibiting inflammation, and regulating the expression of aquaporin–1 (AQP–1) and zonula occluden–1 (ZO–1) in a PDS-induced RPMC model [15]. However, it is not clear that the therapeutic effect of the PPAR-γ agonist on a PD rat model with LPS-induced peritonitis is associated with regulation of AQP1 and ZO-1 expression. The present study was designed to investigate the effect and mechanism of rosiglitazone treatment on the peritoneal alterations in a LPS-induced PD rat model.

## Methods

### Animal model and experimental procedures

Six-week-old male Sprague-Dawley rats (weighing from 215 to 240 g) were purchased from the Laboratory Animal Center of Academy of Military Medical Sciences (Beijing, China). All animal procedures were conformed to the standard operating procedures approved by the Institutional Animal Ethics Committee of Huadu District People’s Hospital, Southern Medical University. The rats were housed in a temperature-controlled environment (22 ± 1°C) under a 12 h light/dark cycle with free access to water and food. After adaptive feeding for 2–3 days, the rats were randomly assigned to five groups as follows: Group A (Control): Normal Rats; Group B (Mock): peritoneal dialysis (PD) + i.p. normal saline; Group C (Model): PD + lipopolysaccharide (LPS); Group D (Rosiglitazone L): PD + LPS + rosiglitazone (15 mg/kg i.p.); Group E (Rosiglitazone H): PD + LPS + rosiglitazone (20 mg/kg i.p.) (*n*=5). The rats were intraperitoneally injected with 15 or 20 mg/kg rosiglitazone (GlaxoSmithKline, Stevenage, U.K.) dissolved in normal saline for consecutively 7 days. Meanwhile, the Mock and Model group were injected with the same solution instead. Before injecting 100 ml/kg PD solution containing 3.86% glucose (Sigma-Aldrich, St. Louis, MO, U.S.A.), rats were intraperitoneally injected with 1 mg/kg LPS (Sigma-Aldrich) to induce inflammatory responses including peritoneal fibrosis, while the mock group was administrated with 1 ml/dl 0.9% normal saline as control. Then, a 90 min peritoneal equilibrium test (PET) was performed with 30 ml of 3.86% PDF. Briefly, a self-made peritoneal dialysis catheter was implanted 2 cm below the ribs of the rat, and the omentum was partially removed during the operation. The peritoneal dialysis catheter exit was 1 cm below the midpoint of the line connecting the ears and the back of the head and neck. If 20 ml of normal saline injected through the peritoneal dialysis catheter can flow out smoothly from the other end, the tube is successfully implanted.

After 90 min, the paracentesis needle was hooked to a vacuum bottle by a small tube for the fluid to drain into. The ultrafiltration volume dialysate-to-plasma urea ratio (D/P urea), dialysate-to-plasma albumin (D/P albumin), glucose reabsorption (D_90_/D_0_ glucose), and inflammatory cells of the dialysate including neutrophil and lymphocyte were determined. Immediately thereafter, the rats were anesthetized with 4% isoflurane and maintained at a level of 2% isoflurane in O_2_. Dialysate samples and blood samples were collected, after which the rats were killed by exsanguinations.

### Histological assessments

Peritoneal tissues samples including visceral peritoneum and parietal peritoneum were obtained from liver and mesentery, then fixed in 10% neutral buffered formalin (NBF). Thereafter, the tissues were paraffin-processed and embedded. The deparaffinized tissue sections (3–5 μm thick) were stained with hematoxylin–eosin (HE) for histological analyses. Meanwhile, the sections of visceral peritoneum and parietal peritoneum were stained with trichrome modified Masson’s stain kit (ScyTek Laboratories, Logan, UT, U.S.A.) to assess fibrosis. The samples were analyzed by light microscopy, combined with Image-Pro Plus (Media Cybernetics, Silver Spring, Maryland, U.S.A.). The thickness of the peritoneum was measured in the ten thickest fields in each liver and mesentery section. The count of vessels was expressed as the mean of vessel numbers in six fields from each liver and six fields from each mesentery peritoneum. Vessel density was expressed as n/mm^2^. Hypercellularity, i.e. proliferation of mesothelial cells or migration of other cells, was scored as: (1) monolayer of cells; (2) double layers; (3) more than two layers in any part of the zone. All images were assessed in a blind manner.

### Western blot analysis

The peritoneal tissue proteins were extracted using T-PER tissue protein extraction reagent added combined protease and phosphatase inhibitors (Thermo Fisher Scientific, Waltham, MA, U.S.A.). The protein concentrations were measured by BCA protein assay kit (Thermo Fisher Scientific) and equal amount of proteins were subjected to SDS-PAGE, then transferred to nitrocellulose membranes (Bio–Rad, Hercules, CA, U.S.A). After blocked with 5% nonfat milk, the membranes were incubated with antibodies against AQP-1 (1:500) (Santa Cruz, Dallas, Texas, U.S.A.), ZO-1 (1:500), PPAR-γ (1:500), p-PPAR-γ (1:500) (Thermo Fisher Scientific), and GAPDH (1:1,000) (Cell Signaling Technology, Beverly, MA, U.S.A.) respectively, followed by incubation with the HRP–conjugated secondary antibodies. Chemiluminescence was detected using ECL kit (GE Healthcare Life Sciences, Pittsburgh, PA, U.S.A.).

### Quantitative real-time RT-PCR

The peritoneal tissue RNA was extracted with RNAprep pure tissue kit (Tiangen Biotech Co., LTD., Beijing, China). The mRNA of AQP-1, ZO-1, and PPAR-γ was amplified by quantitative real-time RT-PCR. It was carried out using SuperScriptIII Platinum SYBR Green One-Step qRT-PCR kit (Invitrogen, Carlsbad, California, U.S.A.) by an ABI PRISM 7500 Fast real-time PCR instrument (Applied Biosystems, Foster City, CA, U.S.A.). The qRT-PCR was carried out with the following procedures: 50°C for 3 min, 95°C for 5 min, followed by 38 cycles of 95°C for 15 s, 60°C for 30 s, with specific primers as follows: AQP-1, forward primer, 5′-TGGCCGAAATGACCTGGCTCG-3′, reverse primer, 5′-GGCGCCTCCGGTCAGTGGTA-3′; ZO-1, forward primer, 5′-AGCGAAGCCACCTGAAGATA-3′, reverse primer, 5′-GATGGCCAGCAGGAATATGT-3′; PPAR-γ, forward primer, 5′-TGATATCGACCAGCTGAACC-3′, reverse primer, 5′-GTCCTCCAGCTGTTCGCCA-3′; GAPDH, forward primer, 5′-AATGCATCCTGCA CCACCA A-3′, reverse primer, 5′-GATGCCATAT TCATTGT CATA-3′. The relative mRNA amounts of AQP-1, ZO-1, and PPAR-γ were calculated by comparative *C*_t_ method.

### Statistical analyses

Statistical analyses were performed by SPSS 20.0 (SPSS, U.S.A.). Data were presented as mean ± SD and analyzed by one-way ANOVA with multiple comparisons test (three or more data sets in a group). *P*<0.05 was considered to indicate statistical significance.

## Results

### Rosiglitazone decreased the number of dialysate neutrophil in LPS-induced PD rat

The characteristics of 25 rats and their dialysate cellular composition were summarized in Table 1. Among the five groups, there were no significant differences in the bodyweight changes and blood glucose levels on day 7. The dialysate neutrophil count in Group D and Group E was lower than that in Group C on day 7, and the inhibitory effect was even more obvious in Group E (Group C: 452 (267–643) vs. Group E: 34 (13–72), *P*<0.01). However, there were basically no differences in the lymphocyte counts among these five groups (Table 1). These results suggested that rosiglitazone could inhibit the production of neutrophils in drained dialysate induced by LPS treatment.

**Table 1:** Characteristics of the rats and dialysate cellular composition

	Group A	Group B	Group C	Group D	Group E
Initial BW (g)	218 (207–238)	215 (206–234)	220 (198–242)	215 (200–230)	216 (202–234)
BW change (g)	65 (50–85)	70 (50–90)	65 (55–80)	80 (50–90)	70 (50–80)
Blood glucose (mg/dl)	208 (184–269)	231 (204–286)	197 (153–253)	223 (173–281)	183 (158–275)
Dialysate neutrophil (/μl)	267 (172–465)	338 (217–584)	452 (267–643)	194 (126–367)	34 (13–72)**
Dialysate lymphocyte (/μl)	194 (72–324)	286 (134–586)	376 (202–647)	341 (197–563)	291 (137–539)

Note: Group A (Control): normal rats; Group B (Mock): peritoneal dialysis (PD) + i.p. normal saline; Group C (Model): PD + lipopolysaccharide (LPS); Group D (Rosiglitazone L): PD + LPS + i.p. rosiglitazone (15 mg/kg); Group E (Rosiglitazone H): PD + LPS + i.p. rosiglitazone (20 mg/kg). BW, bodyweight; **P<0.01 vs. Group C.

### Rosiglitazone improved peritoneal morphology after the LPS administration

**Figure 1:**
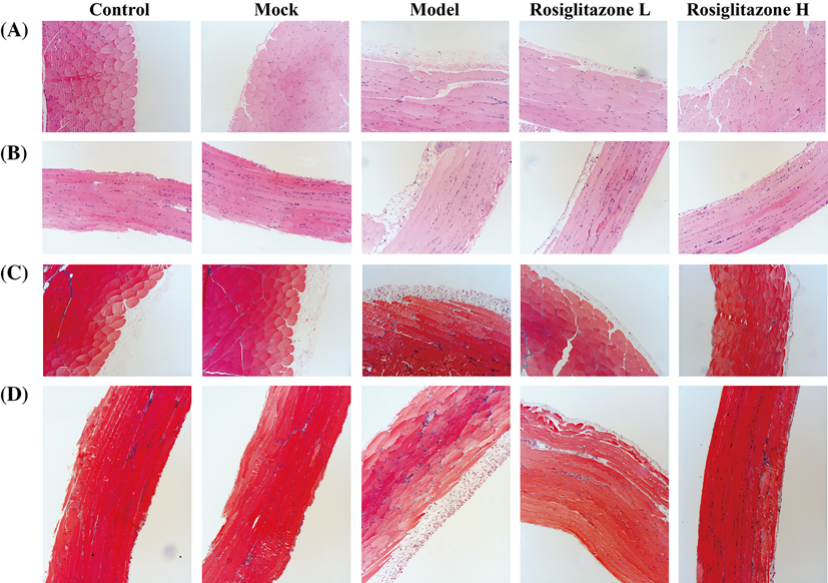
Effects of rosiglitazone on morphological changes of peritoneum (**A** and **B**) Effects of rosiglitazone on morphological changes of parietal peritoneum (A) and visceral peritoneum (B) were determined by H&E staining. (**C** and **D**) Effects of rosiglitazone on morphological changes of parietal peritoneum (C) and visceral peritoneum (D) were determined by Masson staining.

To determine the effect of rosiglitazone on peritoneal morphology after the LPS administration, we performed HE staining and Masson staining for tissue samples including visceral peritoneum and parietal peritoneum. Compared with the Mock group, the Model group lost much of integrity of the mesothelium (Figure 1A,B) and exhibited fibrosis as indicated Masson staining (Figure 1C,D), while the thickness of peritoneal membrane, the density of vessel and the prevalence of hypercellularity were higher in the Mock group (*P*<0.05) (Figure 1 and Table 2). In Rosiglitazone L and Rosiglitazone H treated groups, the integrity of mesothelium and the extent of fibrosis were better compared with the Model group. (Figure 1A,B). Meanwhile, the thickness of peritoneal membrane, the density of vessel, and the prevalence of hypercellularity were reduced in rosiglitazone-treated groups dose-dependently, the restrained trend was especially obvious in Rosiglitazone H group (*P*<0.05) (Table 2).

**Table 2:** Peritoneal morphology (thickness of peritoneal membrane, density of vessel, and scores of hypercellularity)

	Group A	Group B	Group C	Group D	Group E
Peritoneal thickness (μm)	12.6 (10.7–18.6)	11.7 (9.8–17.3)	31.6 (27.5–38.2)^*^	27.3 (24.1–30.6)	15.8 (12.6–20.1)^†^
Vessel density, *n*/mm^2^	64 (12–94)	59 (13–86)	237 (198–322)^*^	176 (152–246)	91 (58–137)^†^
Hypercellularity, *n* (%)					
1	0 (0)	0 (0)	0 (0)	0 (0)	0 (0)
2	2 (40)	1 (25)	0 (0)	1 (25)	4 (80)
3	3 (60)	3 (75)	5 (100)	3 (25)	1 (20)

Note: Group A (Control): normal rats; Group B (Mock): peritoneal dialysis (PD) + i.p. normal saline; Group C (Model): PD + lipopolysaccharide (LPS); Group D (Rosiglitazone L): PD + LPS + i.p. rosiglitazone (15 mg/kg); Group E (Rosiglitazone H): PD + LPS + i.p. rosiglitazone (20 mg/kg). Hypercellularity was scored as: 1, monolayer of cells; 2, double layers, and 3, more than three layers on any part of the zone; *P<0.05 vs. Mock group; ^†^*P*<0.05 vs. Model group.

### Rosiglitazone decreased D/P urea and D/P albumin in LPS-induced PD rats

For the 90 min PET test, there were no statistical difference among the five groups in terms of D_90_/D_0_ glucose (*P*>0.05). Drainage volume in Group C is much smaller than that in Group A and B. Rosiglitazone treatment in Group D and E increased drainage volume compared with Group C. In addition, the D/P urea and D/P albumin in Group C were higher than that in Group B (D/P urea: median 837.6 vs. 715.6, *P*<0.05; D/P albumin: median 14.6 vs. 8.6, *P*<0.05), and Group E had a significantly lower D/P urea (median 735.6 vs. 837.6, *P*<0.05) and D/P albumin (median 9.8 vs. 14.6, *P*<0.05) as compared with Group C (Table 3). The results indicated rosiglitazone restrains albumin and urea (D/P) into peritoneum.

**Table 3:** Peritoneal transport characteristics

	Group A	Group B	Group C	Group D	Group E
Drainage volume (ml)	21 (19.0–23.5)	20.5 (19.0–22.5)	14.5 (11.5–16.0)^*^	16.0 (14.5–18.5)	18.5 (17.0–21.5)^†^
D/P urea x 1000	684.5 (674.6–763.7)	715.6 (643.9–742.8)	837.6 (784.9–952.1)^*^	769.1 (698.3–824.6)	735.6 (657.9–763.4)^†^
D/P albumin x 1000	8.2 (6.5–10.7)	8.6 (6.8–10.3)	14.6 (12.4–16.3)^*^	12.4 (10.8–14.5)	9.8 (8.6–11.7)^^†^^
D_90_/D_0_ glucose	0.36 (0.31–0.42)	0.37 (0.32–.41)	0.34 (0.29–0.43)	0.35 (0.31–0.47)	0.38 (0.31–0.42)

Note: Group A (Control): normal rats; Group B (Mock): peritoneal dialysis (PD) + i.p. normal saline; Group C (Model): PD + lipopolysaccharide (LPS); Group D (Rosiglitazone L): PD + LPS + i.p. rosiglitazone (15 mg/kg); Group E (Rosiglitazone H): PD + LPS + i.p. rosiglitazone (20 mg/kg). D/P urea, dialysate-to-plasma urea ratio; D/P albumin, dialysate-to-plasma albumin ratio; D_90_/D_0_ glucose, ratio of dialysate glucose at 90 min to dialysate glucose at 0 min; *P<0.05 vs. Mock group; ^†^*P*<0.05 vs. Model group.

### Rosiglitazone induced the expression of AQP-1, ZO-1, and p-PPAR-γ in LPS-induced PD rats

**Figure 2:**
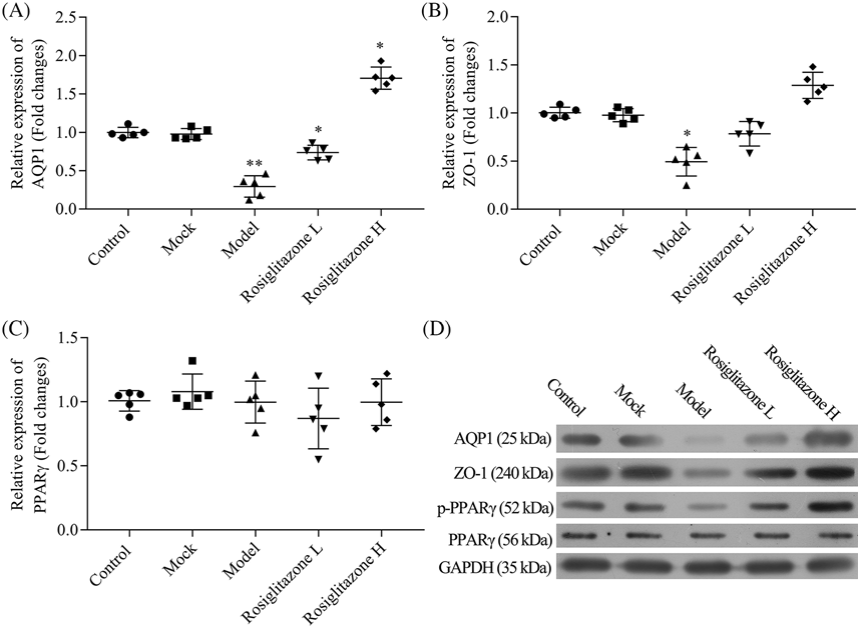
Effects of rosiglitazone on the mRNA and protein expression of AQP–1, ZO–1, and PPAR–γ in LPS-treated peritoneal tissue (**A**–**C**) mRNA levels of AQP–1, ZO–1, and PPAR–γ in peritoneal tissue were determined using real-time PCR analysis. (**D**) Protein expression of AQP–1, ZO–1, PPAR–γ, and p-PPAR–γ in peritoneal tissue were determined by Western blot analysis. Each value represents mean + SD (*n*=5); *P<0.05, **P<0.01 vs. Mock group.

As abnormal expression of AQP-1 and ZO-1 leads to degenerated function of the peritoneum, and abnormal expression of PPAR-γ is related to peritoneal fibrosis and neoangiogenesis, we determined the effect of rosiglitazone on the expression of AQP–1, ZO-1, and PPAR-γ. Compared with the Mock group, the gene expression levels of AQP-1 and ZO-1 showed a significantly decreased trend in Model group (AQP-1: *P*<0.01; ZO-1: *P*<0.05), and rosiglitazone induced the RNA expression of AQP-1 and ZO-1 dose-dependently. Especially, rosiglitazone at high concentration exhibited obvious effect (*P*<0.05) (Figure 2A–C). Consistent with the results of qRT-PCR analysis, the protein expression of AQP-1 and ZO-1 were also significantly reduced in the Model group, as demonstrated by the Western blot analysis. While rosiglitazone up-regulated the protein expression of AQP-1 and ZO-1 in a dose-dependent manner (Figure 2D).

On the other hand, for the PPAR-γ expression analysis, there were no significant differences on total RNA and protein expression of PPAR-γ among these groups. However, rosiglitazone induced the protein expression of p-PPAR-γ compared with Mock group, which was inhibited by LPS treatment (Figure 2A–D).

## Discussion

Peritonitis represents a common and severe complication in patients with PD. Despite the significant decline of the peritonitis rates in recent years, peritonitis is still the major cause of hospitalization, catheter loss, and technique failure, and even the death in PD patients [16–18].

Clinical data show that the structure and function of the peritoneal membrane in PD patients is severely impaired during peritonitis [19]. Meanwhile, accumulated evidence demonstrated that the peritoneal membrane in patients with acute peritonitis showed the increased transport of small molecular solutes and decreased ultrafiltration [20,21]. In the present study, we evaluated LPS-induced peritonitis model.

Unfortunately, it is still lack of strategy to prevent or treat the alteration of structure and function of the peritoneal membrane caused by peritonitis. In the present study, our aim is to evaluate the effects of PPAR-γ agonist, rosiglitazone, on the peritoneum of LPS-induced rat receiving peritoneal dialysis.

Consistent with our and other previous findings [14,22], LPS treatment significantly increased the number of dialysate neutrophil and lymphocyte in rat treated with PD, suggesting that the symptom of this model is in part similar with that of the peritonitis. However, rosiglitazone dose-dependently decreased the number of dialysate neutrophil in LPS-induced rat treated with PD, which suggests that rosiglitazone inhibits the inflammatory response. This can be explained by the fact that rosiglitazone is an agonist of PPAR-γ which has been shown anti-inflammatory role. However, this finding was somewhat different from the previous study showing that rosiglitazone did not decrease dialysate lymphocyte [22]. We believe that this difference is resulted from dose difference of rosiglitazone used between our study and the previous study. In our previous study, we have shown that rosiglitazone attenuates inflammation via NF-κB inhibition and supplied protective effects in LPS-induced peritonitis rats [14]. Therefore, rosiglitazone-mediated inhibition of inflammation might be one of major contributors to the improved peritoneal morphology in LPS-treated rat with PD, which is reflected by the reduced thickness of peritoneal membrane, the reduced density of vessel, and the reduced prevalence of hypercellularity in LPS-induced rat receiving rosiglitazone.

Interestingly, in the present study we found that rosiglitazone dose-dependently increased the drainage volume in LPS-induced rats, which is not correlated with D_90_/D_0_ glucose since D_90_/D_0_ glucose did not differ between rosiglitazone-treated rats and rosiglitazone-untreated rats. A possible reason is that treatment of rosiglitazone influenced aquaporin (AQP)-mediated water transport in the present study. AQPs are a group of water-selective channel proteins, which play an important role in maintaining fluid balance in an organism. AQP-l was the first identified member and has been shown to regulate osmotically driven water movement across the peritoneum [23]. The deletion of AQP-1 has been shown to change the structure of the mouse peritoneum and lead to solute-free ultrafiltration decreased by ~70% and cumulative decreased by almost 50% [24]. Furthermore in our previous study, rosiglitazone has been shown to up-regulate AQP-1 expression and subsequently resulted in the increased reabsorption of water and sodium a PDS-induced RPMC model [15]. Similarly, our present study also demonstrated that rosiglitazone dose-dependently increased the expression of AQP-1 in the peritoneum of LPS-induced peritonitis rats.

In addition, improved function of the peritoneum in LPS-induced PD rats receiving rosiglitazone, which is reflected by improved D/P urea and D/P albumin, is possibly associated with increased ZO-1 expression. ZO-1, also known as tight junction protein-1, is a peripheral membrane protein that is encoded by the TJP1 gene in humans. ZO-1 functions to maintain epithelial cell polarity and is indirectly involved in the formation of the cytoskeleton. In the mesothelial monolayer, different intercellular junctions hold mesothelial cells to each other. The intercellular junctions can dynamically alter their structural and functional properties under different conditions and can be modulated by various cellular and metabolic regulators [25]**.** Therefore, ZO-1 plays an important role in the integrity of the structure and function of the peritoneum. In the present study, we found that LPS treatment significantly reduced ZO-1 expression of the peritoneum in PD rats. However, rosiglitazone dose-dependently increased ZO-1 expression of the peritoneum in PD rats treated with LPS. This present finding is similar to our previous study showing that the rosiglitazone increased ZO-1 expression in the rat peritoneal mesothelial cells treated with 4.25% peritoneal dialysis solution [15]. Thus, rosiglitazone improved the damaged structure and function of the peritoneum in LPS-induced PD rats at least partly through up-regulation of AQP-1 and ZO-1.

Of note, we did not observe changed expression of AQP1 and ZO-1 expression between the control group (normal mice without treatment of PD and LPS) and Mock group (mice with PD not LPS treatment) in the present study. This is different from decreased expression of AQP1 and ZO-1 in RPMCs treated with PD. A possible reason is that treatment of 90 min-PD in normal rats is not enough to change AQP1 and ZO-1 expression. Conversely, 90 min-PD treatment in the LPS-induced peritonitis rats is enough to decrease AQP1 and ZO-1 expression. These findings suggest that failure of PD in patients with peritonitis caused by bacterial infection might be associated with quickly decreased AQP1 and ZO-1 expression. However, further study is needed to exactly clarify this relevance.

In conclusion, we show that administration of rosiglitazone provides pleiotropic protective effects on the peritoneal membrane to LPS-induced mice exposed to PD fluid, by reducing inflammation and preserving mesothelial cell monolayer resulted from up-regulation of AQP-1 and ZO-1. Our findings shown that PPAR-γ activation might be a reasonable strategy to prevent and ameliorate peritoneal deterioration in PD patients, especially with peritonitis.

## Abbreviations

HE: hematoxylin–eosin
LPS: lipopolysaccharide
NBF: neutral buffered formalin
PD: peritoneal dialysis
PET: peritoneal equilibrium test
PMC: peritoneal mesothelial cells
PPAR-γ: peroxisome proliferator-activated receptor γ
UF: ultrafiltration
UFF: ultrafiltration failure

## References

[1] Ditsawanon P. and Aramwit P. (2015) Preserving the peritoneal membrane in long‐term peritoneal dialysis patients. J. Clin. Pharm. Ther. 40, 508–516 10.1111/jcpt.12318 26280248

[2] Yuvaraj A. (2015) Diagnostic dilemma of ultrafiltration failure in a continuous ambulatory peritoneal dialysis patient. Perit. Dial. Int. 35, 233–234 10.3747/pdi.2014.00086 25904776 PMC4406321

[3] Teitelbaum I. (2015) Ultrafiltration failure in peritoneal dialysis: a pathophysiologic approach. Blood Purif. 39, 70–73 10.1159/000368972 25661912

[4] Aroeira L.S. (2007) Epithelial to mesenchymal transition and peritoneal membrane failure in peritoneal dialysis patients: pathologic significance and potential therapeutic interventions. J. Am. Soc. Nephrol. 18, 2004–2013 10.1681/ASN.2006111292 17568021

[5] Ranzinger J., Rustom A. and Schwenger V. (2014) Membrane nanotubes between peritoneal mesothelial cells: functional connectivity and crucial participation during inflammatory reactions. Front. Physiol. 5, 412, 10.3389/fphys.2014.00412 25386144 PMC4208614

[6] Morelle J. and Devuyst O. (2015) Water and solute transport across the peritoneal membrane. Curr. Opin. Nephrol. Hypertens. 24, 434–443 10.1097/MNH.0000000000000151 26197201

[7] Kratochwill K. (2009) Stress responses and conditioning effects in mesothelial cells exposed to peritoneal dialysis fluid. J. Proteome Res. 8, 1731–1747 10.1021/pr800916s 19231869

[8] Mateijsen M. (1999) Vascular and interstitial changes in the peritoneum of CAPD patients with peritoneal sclerosis. Perit. Dial. Int. 19, 517–525 10641771

[9] Riesenhuber A. (2011) Peritoneal dialysis fluid induces p38-dependent inflammation in human mesothelial cells. Perit. Dial. Int. 31, 332–339 10.3747/pdi.2009.00206 21193553

[10] Kowalewska P.M., Margetts P.J. and Fox-Robichaud A.E. (2016) Peritoneal dialysis catheter increases leukocyte recruitment in the mouse parietal peritoneum microcirculation and causes fibrosis. Perit. Dial. Int. 36, 7–15 10.3747/pdi.2014.00211 26475840 PMC4737560

[11] Wang L. (2014) Natural product agonists of peroxisome proliferator-activated receptor gamma (PPARγ): a review. Biochem. Pharmacol. 92, 73–89 10.1016/j.bcp.2014.07.018 25083916 PMC4212005

[12] Choo J. (2015) A novel peroxisome proliferator-activated receptor (PPAR) γ agonist 2-hydroxyethyl 5-chloro-4, 5-didehydrojasmonate exerts anti-inflammatory effects in colitis. J. Biol. Chem. 290, 25609–25619 10.1074/jbc.M115.673046 26342083 PMC4646205

[13] Peng Y. (2006) Troglitazone inhibits synthesis of transforming growth factor‐β1 and reduces matrix production in human peritoneal mesothelial cells. Nephrology 11, 516–523 10.1111/j.1440-1797.2006.00654.x 17199790

[14] Zhang Y.-F. (2015) Rosiglitazone, a peroxisome proliferator-activated receptor (PPAR)-γ agonist, attenuates inflammation via NF-κB inhibition in lipopolysaccharide-induced peritonitis. Inflammation 38, 2105–2115 10.1007/s10753-015-0193-2 26047949

[15] Zhang Y.F. (2017) PPAR-γ agonist rosiglitazone protects rat peritoneal mesothelial cells against peritoneal dialysis solution-induced damage. Mol. Med. Rep. 15, 1786–1792 10.3892/mmr.2017.6196 28259952

[16] Group C.-U.P.D.S. (1996) Adequacy of dialysis and nutrition in continuous peritoneal dialysis: association with clinical outcomes. J. Am. Soc. Nephrol. 7, 198–207 8785388 10.1681/ASN.V72198

[17] Boudville N. (2012) Recent peritonitis associates with mortality among patients treated with peritoneal dialysis. J. Am. Soc. Nephrol. 23, 1398–1405 10.1681/ASN.2011121135 22626818 PMC3402287

[18] Li P.K.-T. (2016) ISPD peritonitis recommendations: 2016 update on prevention and treatment. Perit. Dial. Int. 36, 481–508 10.3747/pdi.2016.00078 27282851 PMC5033625

[19] Dobbie J.W. (1994) Ultrastructure and pathology of the peritoneum in peritoneal dialysis. In The Textbook of Peritoneal Dialysis, pp. 17–44, Springer

[20] Pollock C. (1989) Loss of ultrafiltration in continuous ambulatory peritoneal dialysis (CAPD). Perit. Dial. Int. 9, 107–110 2488194

[21] Verger C. (1983) Acute changes in peritoneal morphology and transport properties with infectious peritonitis and mechanical injury. Kidney Int. 23, 823–831 10.1038/ki.1983.101 6887693

[22] Song S.H. (2009) Role of rosiglitazone in lipopolysaccharide‐induced peritonitis: a rat peritoneal dialysis model. Nephrology 14, 155–163 10.1111/j.1440-1797.2008.01037.x 19207869

[23] Devuyst O. and Yool A.J. (2010) Aquaporin-1: new developments and perspectives for peritoneal dialysis. Perit. Dial. Int. 30, 135–141 10.3747/pdi.2010.00032 20200365

[24] Ni J. (2006) Aquaporin-1 plays an essential role in water permeability and ultrafiltration during peritoneal dialysis. Kidney Int. 69, 1518–1525 10.1038/sj.ki.5000285 16508653

[25] Nusrat A., Turner J. and Madara J. IV (2000) Regulation of tight junctions by extracellular stimuli: nutrients, cytokines, and immune cells. Am. J. Physiol. Gastrointest. Liver Physiol. 279, G851–G857 10.1152/ajpgi.2000.279.5.G851 11052980

